# Risk Perception and Worries among Health Care Workers in the COVID-19 Pandemic: Findings from an Italian Survey

**DOI:** 10.3390/healthcare8040535

**Published:** 2020-12-03

**Authors:** Mariangela Valentina Puci, Guido Nosari, Federica Loi, Giulia Virginia Puci, Cristina Montomoli, Ottavia Eleonora Ferraro

**Affiliations:** 1 Unit of Biostatistics and Clinical Epidemiology, Department of Public Health, Experimental and Forensic Medicine, University of Pavia, 27100 Pavia, Italy; mariangela.puci@unipv.it (M.V.P.); giuliapuci0193@gmail.com (G.V.P.); cristina.montomoli@unipv.it (C.M.); 2 Department of Neurosciences and Mental Health, Fondazione IRCCS Ca’ Granda Ospedale Maggiore Policlinico, 20100 Milan, Italy; guido.nosari@gmail.com; 3 Regional Epidemiological Veterinary Observatory, Istituto Zooprofilattico Sperimentale della Sardegna G. Pegreffi, 07100 Cagliari, Italy; federica.loi@izs-sardegna.it

**Keywords:** COVID-19, health-care workers, risk perception

## Abstract

The ongoing pandemic scenario, due to the coronavirus disease 2019 (COVID-19), has had a considerable impact on public health all over the world. Italy was one of the most affected countries, as the first European full-blown outbreak occurred there. The exposure of the Italian health care workers to COVID-19 may be an important risk factor for psychological distress. The aim of this cross-sectional study was to describe worries and risk perception of being infected among Italian Health Care Workers (HCWs) during the first wave of the pandemic. In total, 2078 HCWs participated in a web survey (78.8% were females). The highest percentage of respondents were physicians (40.75%) and nurses (32.15%), followed by medical (18.00%), health care support (4.50%) and administrative (4.60%) staff. In a score range between 0 (not worried) and 4 (very worried), our results showed that participants declared that they were worried about the Coronavirus infection with a median score of 3 (IQR 2-3) and for 59.19% the risk perception of being infected was very high. In addition, HCWs reported they suffered from sleep disturbances (63.43%). From the analysis of the psychological aspect, a possible divergence emerged between the perceived need for psychological support (83.85%) and the relative lack of this service among health care providers emerged (9.38%). Our findings highlight the importance of psychological and psychiatric support services not only during the COVID-19 pandemic, but also in other emerging infectious diseases (EIDs) scenarios. These services may be useful for health authorities and policymakers to ensure the psychological well-being of health care professionals and to promote precautionary behaviors among them.

## 1. Introduction

Over the past decades, the onset of new highly infectious diseases has caused severe public health problems affecting millions of people around the world [1]. As defined by the World Health Organization (WHO), emerging infectious diseases (EIDs) are infections that either first appeared in a population, or were already present but rapidly increased in the number of new cases, or spread in a new geographic area [2]. Most of the EIDs are zoonotic, that is diseases naturally transmitted from animals to humans [3,4], and their origins are significantly associated with environmental and socioeconomic factors [5,6,7].

Among the best-known EIDs are the Severe Acute Respiratory Syndrome (SARS), Ebola, HIV/AIDS, the Nipah virus encephalitis, avian and H1N1 influenza and the Middle East respiratory syndrome (MERS) coronavirus [2,8]. Unfortunately, many of these diseases still do not have a specific licensed vaccine [1], and health care workers (HCWs) are often victims of them. Moreover, HCWs are not only at a high risk of infection, but they may also contribute to their spread from hospital to community environments and vice versa [9,10].

A stressful workplace has detrimental effects on both the quality of life and the professional activity of HCWs, in terms of limited efficiency and ability to provide high-quality health services [11,12]. Furthermore, during an emergency such as either an epidemic or a pandemic, HCWs can develop psychological distress and concerns about the surrounding people’s health [13,14]. Therefore, the benefits provided and the performance given by workers responsible for health services may largely depend on their skills, knowledge and motivation [15].

In regard to EIDs, the spread of the severe acute respiratory syndrome coronavirus (COVID-19), caused by the SARS-CoV-2 virus with the first cases reported last December in Wuhan (China), has reached pandemic proportions with severe physical and mental health consequences [16] and over 62 million confirmed cases [17]. Since the detection of the first case on 22 February 2020 and during the first wave of the pandemic period, Italy has been one of the most affected countries in Europe with more than two hundred thousand infected people [18]. Currently, the Italian epidemiological context is still alarming: at the date of 1 December 2020, there are more than 1.6 million of confirmed cases and more than 55 thousand deaths [17]. As a result, from 9 March to 3 May the whole country was subjected to strong lockdown measures aimed at controlling the spread of the disease. During that period, and since 4 May (the new Italian phase of reopening and gradual easing of the restrictions, when manufacturing activities were reopened), hospitals, nursing homes and therapeutic communities (TCs) have had the primary role in the patient’s care. Italian HCWs have worked in the highest intensity scenario of the past 30 years and it is publicly recognized that during the pandemic scenario HCWs played a crucial role in the management of health emergency, despite high personal risks and they were psychologically and physically burned out because of the workload of those months [19].

For this reason, our study aims to describe worries and risk perception of being infected of the Italian HCWs working either in hospitals, nursing homes or therapeutic communities during the first wave of the COVID-19 pandemic.

## 2. Materials and Methods

### 2.1. Study Design and Participants

This is a cross-sectional study using a web-based open survey. The study population included physicians, nurses, medical staff (radiologic technologists, rehabilitation technicians, physiotherapists and midwives), health care support and administrative personnel working in hospitals, nursing homes and therapeutic communities in Italy during the first wave of the pandemic period (February–May 2020). Trainee students, who had not obtained the qualification yet at the time of the interview, could not participate in the study. All participants, who had been recruited by using a “snow-ball” sampling, by email, newsletters and social media (Facebook and LinkedIn), were required to provide an informed consent before taking part in the web survey and to agreeing to data collection and storage for analysis and publication. The survey was completely anonymous. Participants were aware of the voluntary nature of their participation and confidentiality of information was assured. The final sample size was equal to the number of HCWs who completed the online questionnaire and met the inclusion criteria (that is, being a health professional with the necessary qualifications and providing informed consent). The study received the approval for investigation from the Ethics Committee.

### 2.2. Data Collection

We drew up an online questionnaire using the online Google platform “Form” and it was available online from 5 May to 6 June.

In this self-reported questionnaire, the following information was collected: age, sex, marital status, region of residence (recoded in geographic area: North, Centre and South), work experience (expressed in years), educational level, occupational category and hospital ward/department, perceived risk of infection (high, medium, or low), worries and knowledge about the pandemic. Items about worries over COVID-19 had a score that ranged between 0 to 4, where 4 represents the highest degree of concern.

### 2.3. Statistical Analysis

The completeness and consistency of the collected data, which were stored in an ad hoc database, were evaluated. Quantitative variables were summarized as mean values, standard deviations (sd), median and interquartile range (IQR), whereas qualitative variables were summarized as frequencies and percentage. In order to compare qualitative variables, either the Chi-square test or the Fisher exact test was applied. In order to compare differences between quantitative variables the Kruskal–Wallis nonparametric test was applied. The level of *p* < 0.05 was considered significant for all the analysis, except for multiple comparisons, for which the Bonferroni correction was used (*p* value/n of contrast in the five groups: *p* < 0.01). The software employed to carry out the analysis was STATA/SE for Window, version 15 (StataCorp., College Station, TX, USA).

## 3. Results

### 3.1. Participants and Socio-Demographic Characteristics

In total, 2103 subjects participated in the survey; among these, 2078 met the inclusion criteria. Table 1 shows the participants’ socio-demographic and professional characteristics.

Among 2078 health care workers, the mean age was 42 ± 11 years. In total, 1637 (78.80%) were females. The highest percentage of respondents were physicians (40.75%) and nurses (32.15%), followed by medical (18.00%), health care support (4.50%) and administrative (4.60%) staff.

### 3.2. Worries about the Coronavirus Pandemic

A total of 15.80% reported having been infected with SARS-CoV-2. Table 2 shows the items related to worries about COVID-19. 

In the sample, the most frequent worry was about “The risk of infection for the surrounding people” (A.1.3): the percentage ranged from 53.68% (administrative staff) to 55.84% (nurses). Only a limited number of subjects (0.87%) reported having no worries. Physicians (4.84%) appeared to be more worried about “The isolation from family and-or social environment” (A.1.4), whereas administrative staff (24.21%) were upset about “The disease’s dangerousness/health consequences” (A.1.2). Participants declared that they were worried about the COVID-19 infection (A.3) with a median score of 3 (IQR 2–3). Similar results are shown for health care professional groups: post hoc analysis reveals significant differences between administrative staff and health care support personnel (Bonferroni post hoc test, *p* = 0.0015) and between administrative staff and nurses (Bonferroni post hoc test, *p* = 0.003).

With regard to the lack of information provided by the department/ward (A.5.1) to each professional group, only 1/5 of the subjects were very concerned about it. Furthermore, the percentage of participants who declared themselves to be very worried about the shortage of personal protective equipment (A.5.2) and about intentional absenteeism (A.5.4) was higher in the health care support staff group than in the other health care workers groups (39.36% and 17.02%, respectively, *p* < 0.001). Finally, only few HCWs reported their concern about the lack of recognition for their work (percentages ranged from 3.90% for physicians to 8.56% for medical personnel).

### 3.3. Perceived Risk of Infection and Information about the COVID-19 Pandemic

Table 3 shows the results regarding the perceived risk of being infected and the related information. At the beginning of the pandemic scenario, 59.19% of the HCWs considered the risk of being infected very high (B.1), but with differences among the groups (*p* = 0.001): physicians and nurses regarded it as higher compared to other HCWs (60.92% and 62.87%, respectively). The lowest risk of infection (45.26%) was perceived by the administrative staff.

For all HCWs groups, when the pandemic scenario started, the perception of risk of being infected was significantly higher in the workplace (B.2.) than in the outside environment (*p* < 0.001), with percentages that ranged between 59.89% (for medical staff) and 81.11% (for physicians).

Among all health care professionals, the percentage of subjects who believed that the risk of infection was very high and it was still large at the time of the questionnaire administration (B.3.).

Only within the administrative staff group there was an increase in the number of subjects for whom the risk of infection was higher compared to the beginning of the pandemic (from 45.26% to 57.89%).

At the time of the questionnaire administration, workplace was the highest risk environment (B.4, 48.12%). In addition to this, HCWs reported that among the surrounding people (B.5), the infection had mostly spread among their colleagues (35.98%).

The degree of HCWs’ perceived sufficiency of information (B.6) about contamination routes, preventive measures, symptoms, treatment, prognosis and risk factors may be considered moderately high (median score ranged from 4 to 5). However, administrative staff reported a slightly lower median value for treatment and prognosis (3, IQR 2–4 for both) compared to other professionals (*p* = 0.029 and *p* = 0.150, respectively).

Thus, HCWs agreed that the departments/wards proved to be adequately prepared to cope with the emergency due to the infection (B.7) (median 3, IQR 2–4) and that the information they provided (B.8) was complete and comprehensive (median 3, IQR 2–4). In the last two cases, administrative staff reported higher median values (both 4, IQR 3–4) then the other groups: in regard to the information provided by departments/wards, post hoc analysis reveals that differences were significant only in comparison with nurses (Bonferroni post hoc test, *p* = 0.0036) and physicians (both Bonferroni post hoc test, *p* < 0.001).

### 3.4. Psychological Aspects

Table 4 reports the data collected about the psychological aspect. When asked if in their workplace adequate attention was paid to employees’ mental health (C.1), HCWs reported a median value of 3 (IQR 1–3), except for physicians who recorded a median value of 2 (IQR 1–3), which seems to indicate a lower degree of agreement. HCWs reported they suffered from sleep disturbances in the last two months (C.2): the proportion of subjects with sleep disturbances was significantly the highest in the nurses group (70.36%) and the lowest in the administrative staff group (51.58%) (*p* < 0.001).

According to 83.35% of the participants, it was useful to have psychological support during the pandemic scenario (C.3), but only 9.38% of the respondents received it (C.4). The highest proportion is represented by physicians and nurses (10.63% and 10.18%, respectively), the lowest by administrative staff (4.21%). Within all the groups, 38.16% reported that their department/ward did not provide this service (C.5).

Additionally, we observed similar results as to the use of drugs (C.6): the proportion of subjects who used drugs was higher among physicians and nurses than within the other groups (13.70% and 11.23%, respectively, *p* = 0.003). In regard to the type of drugs, the highest percentage was related to the use of anxiolytics, followed by sleeping pills and antidepressants (data not shown).

## 4. Discussion

This study evaluated worries, risk perception and psychological aspects of HCWs during the first COVID-19 pandemic peak in Italy, in the period between February and May 2020. During the emergency, the National Healthcare System’s capacity faced great pressure, since a large number of infected patients with respiratory complications related to COVID-19 required hospitalizations in Intensive Care Units [20]. In order to strengthen the Healthcare System’s capability to deal with the emergency, the Italian Government has allocated funds to increase the number of beds in ICUs and the production of personal protective equipment, and activated a procedure for the immediate recruitment of biologists, physicists, chemists, military doctors, physicians, and nurses [21].

To the best of our knowledge, this is the first study aimed at evaluating these characteristics of Italian HCWs working not only in the hospitals but also in nursing homes and therapeutic communities during the first COVID-19 pandemic peak.

Our study was based on a comprehensive sample of health-related occupational categories (including physicians, nurses, medical personnel, health care support staff, and administrative staff).

Results describe the extent of HCWs concerns about the COVID-19 pandemic (more than half of the HCWs reported being highly worried about this scenario) and offer noticeable insights on rising concerns among the occupational categories involved—albeit to a different extent—about the management of this international health crisis.

As reported in previous studies [22,23,24], participants were mostly females.

As reasonably expected, the perceived risk was significantly higher for physicians and nurses than for other health care professionals, which is consistent with the direct biological exposition to SARS-CoV-2 associated with their bedside activity. The concern for the risk of infection among administrative staff was a little less considerable in comparison with the aforementioned professional categories. It is reasonable to hypothesize that this different level of concern is related to their profession, since the workplace has been identified as the highest top-score degree of infection risk. The assessment of risk perception is central in the EIDs control: a realistic risk perception allows to implement and promote voluntary preventive behaviors that are often the only defense when there is neither an official protocol for treatment nor a licensed vaccine [25].

In our sample risk perception, at the time of the questionnaire administration, (Italian reopening phase) may be hypothesized to be as high as at the beginning of the pandemic.

This result may be due to the way and the intensity of the communication offered by mass media, which may have amplified the information on the risk and, probably, on its perception [25].

Moreover, the emotional and psychological distress related to the dramatic outbreak of the disease during the previous months is likely to have produced a “long wave” of uncertainty among the HCWs who have been the hardest hit subjects.

These results suggest that health care workers’ concerns were diverse and more closely related to their work environment than to their everyday life.

Our results offer some substantial hints for reflection. We may hypothesize that HCWs’ professional status may have determined a significant deal of stress or may have triggered psychological/psychiatric disorders, thus defining this as a “high-risk category” for mental health issues. These topics may be possibly addressed by dedicated trials.

More specifically, many health care professionals of our sample complained about sleep disturbances and/or required psychiatric medications. Although the present study did not examine the nature of the complaints about their mental health reported by health care professionals during the pandemic, it is not unlikely that a large percentage suffered from a new onset of sleep disturbances or a significant worsening of mood. This result may be plausible as a proportion of HCWs reported taking psychotropic medications. In this case as well the percentage of drug users was significantly higher among the HCWs directly involved in bedside activities, such as physicians and nurses.

However, this degree of concern does not appear to stem from a lack of information or training. Perceived sufficiency of information on different aspects of the COVID-19 outbreak was moderately high, and, generally speaking, information on disease treatment and prognosis was clear enough for medical staff, although the score proved to be lower for administrative staff with lower median values. Furthermore, respondents within the different HCWs groups were quite in agreement on the adequate preparedness of the department/ward to cope with the emergency and on the information provided during the pandemic scenario. It is remarkable that, in regard to the last two items, administrative staff reported higher median values than nurses and physicians. This finding is likely to be consistent with the two groups’ different areas of expertise, being the former more focused on service planning and the latter on patient assistance.

Our findings are consistent with those of similar recent studies investigating mental health issues among HCWs in Wuhan, Hubei, during the 2019 Coronavirus outbreak [23,24]. These studies highlight that female nurses and frontline workers from the urban area of Wuhan reported more severe symptoms in all the considered measurements than those ones from different regions. Consistent with our findings, “frontline” work (i.e., nurses and physicians) has been indicated as an independent risk factor for worsening mental health prognosis. These conclusions about the stress level caused by the risk perception are similar to those found in the recent study by Garzaro et al., 2020, which showed and demonstrated that physicians are the leading source of infection as well as one of the most vulnerable groups [26].

Therefore, the present study confirms the emotional fragility of HCWs involved in the acute COVID-19 outbreak. Unlike the ones conducted in Wuhan in 2019, our study underlines a high degree of concern about the pandemic with a transversal dimension throughout all the working categories involved in health assistance (physicians, nurses, technical and administrative staff).

Another striking finding emerging from the questionnaire administration is the evident divergence between the perceived need for psychological support and the relative lack of this service among health care providers. This result may play a key role in forming a spiral of chronicity in the previously cited mental health issues (i.e., insomnia). Starting from the percentage of subjects who gave positive answers, more comprehensive attention should be paid to HCWs’ mental health through dedicated services and constant monitoring of their psychological well-being, also to prevent the risk of burnout syndromes [19]. It is necessary to notice that acknowledging the need for psychological help may still represent a taboo for modern society, and mental illness-related stigma pervades the health care sector and has a strong impact on HCWs’ psychological well-being [27]. In any case, it is arguable that health care workers experiencing psychological distress are likely to face a two-level barrier: the traumatic experience itself and the perspective of the stigma of the working entourage [28].

This study should be interpreted in view of some limitations and bias mainly due to its nature, i.e., cross-sectional survey, where information and answers were self-reported [29]. Another limitation is the lack of a follow-up evaluation, which is necessary to describe and investigate the medium-term psychological implications for HCWs, as they may have suffered significant deterioration, both psychologically and physically. Other limitations are related to the use of non-validated questionnaires and of non-representative sample of the overall Italian health care workers.

Finally, we cannot rule out the possibility that health professionals in deep distress were underrepresented, as these individuals may have been either on leave due to their concerns about the pandemic or extremely busy working in the Intensive Care Unit and consequently unable to join the study.

## 5. Conclusions

The present work points to an important issue related to the impact that the COVID-19 pandemic has on HCWs’ psychological and physical well-being. In particular, it clearly emerges that in an emergency context, the subjects involved can feel worried not only for themselves but also for the surrounding people.

Our findings highlight and suggest the importance of psychological and psychiatric support services in an EIDs scenario, as well as stressing the institutions’ lack of attention to these issues. Managers and team leaders play a leading role in ensuring the psychological well-being of their employees: HCWs in good psychological health during the emergency will be more likely to fulfil their duties. Therefore, it is essential to increase psychosocial support and to facilitate access to it, and to ensure that staff are aware of the possibility of having benefit from these services, with direct or remote access, in order to deal with the needs of hospital shifts. It is a well-known fact that untreated psychic distress leads to even severe health issues, involving both psychiatric and organic diseases, thus compromising work quality and proficiency.

A mentally healthy workplace is achievable in all organizational contexts; however, in order for this to happen, an authentic and continuous commitment is required at all levels. Finally, managers and stakeholders play a key role in risk communication in the workplace, and they can promote precautionary behaviors among health care professionals.

## Figures and Tables

**Table 1:** Participants’ characteristics (*N* = 2078).

Participants Characteristics	All Sample
	***N* = 2078**
Age (years), mean (sd)	42.17 (10.98)
Years of work experience, mean (sd)	15.99 (10.94)
Sex, *n* (%)	
Female	1637 (78.80)
Marital status, *n* (%)	
Single	519 (24.96)
Divorced	139 (6.70)
Widowed	11 (0.53)
Married	1409 (67.81)
Geographic area, *n* (%)	
North	1454 (70.00)
Center	254 (12.20)
South	370 (17.80)
Education, *n* (%)	
High School Diploma	246 (11.80)
Undergraduate Degree	935 (45.00)
Postgraduate Education	897 (43.20)
Type of Health Structure, *n* (%)	
Therapeutic Community	91 (4.40)
Nursing home	145 (7.00)
Hospital	1842 (88.60)
Occupational category, *n* (%)	
Physicians	847 (40.75)
Nurses	668 (32.15)
Medical staff	374 (18.00)
Health care support staff	94 (4.50)
Administrative staff	95 (4.60)

**Table 2:** Health care workers’ concerns and worries about COVID-19 pandemic.

(A) Worries	All *N* = 2078	1. Physicians *N* = 847	2. Nurses *N* = 668	3. Medical Staff *N* = 374	4. Health Care Support Staff *N* = 94	5. Administrative Staff *N* = 95	*p*-Value
(A.1) How much you worry about, *n* (%):							
(A.1.1) The contagion							0.198 #
Very worried	566 (27.24)	220 (25.97)	193 (28.89)	102 (27.27)	27 (28.72)	24 (25.26)	
Quite worried	1292 (62.18)	526 (62.10)	425 (63.62)	227 (60.70)	56 (59.57)	58 (61.05)	
Not worried	220 (10.59)	101 (11.92)	50 (7.49)	45 (12.03)	11 (11.70)	13 (13.68)	
(A.1.2) The disease’s dangerousness/health consequences							0.592 #
Very worried	648 (31.18)	261 (30.81)	206 (30.84)	125 (33.42)	29 (30.85)	27 (28.42)	
Quite worried	1247 (60.01)	500 (59.03)	414 (61.98)	220 (58.82)	55 (58.51)	58 (61.05)	
Not worried	183 (8.81)	86 (10.15)	48 (7.19)	29 (7.75)	10 (10.64)	10 (10.53)	
(A.1.3) The risk of infection for the surrounding people							0.683 #
Very worried	1268 (61.02)	516 (60.92)	408 (61.08)	228 (60.96)	55 (58.51)	61 (64.21)	
Quite worried	724 (34.84)	295 (34.83)	237 (35.48)	132 (35.29)	32 (34.04)	28 (29.47)	
Not worried	86 (4.14)	36 (4.25)	23 (3.44)	14 (3.74)	7 (7.45)	6 (6.32)	
(A.1.4) The isolation from family and/or social environment						0	0.037 #
Very worried	586 (28.20)	258 (30.46)	201 (30.09)	80 (21.39)	25 (26.60)	22 (23.16)	
Quite worried	1018 (48.99)	394 (46.52)	322 (48.20)	201 (53.74)	53 (56.38)	48 (50.53)	
Not worried	474 (22.81)	195 (23.02)	145 (21.71)	93 (24.87)	16 (17.02)	25 (26.32)	
(A.2) What worries you most about, *n* (%):							0.148 #
The contagion	71 (3.42)	29 (3.42)	21 (3.14)	15 (4.01)	3 (3.19)	3 (3.16)	
The risk of infection for the surrounding people	1142 (54.96)	463 (54.66)	373 (55.84)	204 (54.55)	51 (54.26)	51 (53.68)	
The isolation from family and-or social environment	72 (3.46)	41 (4.84)	17 (2.54)	10 (2.67)	1 (1.06)	3 (3.16)	
The disease’s dangerousness/health consequences	338 (16.27)	144 (17.00)	95 (14.22)	62 (16.58)	14 (14.89)	23 (24.21)	
None of the above	18 (0.87)	7 (0.83)	5 (0.75)	3 (0.80)	3 (3.19)	0 (0.00)	
All of the above	437 (21.03)	163 (19.24)	157 (23.50)	80 (21.39)	22 (23.40)	15 (15.79)	
(A.3) How would you score your degree of worry? Score 0–4							<0.001 §
I’m not worried—I’m very worried, mean ± sd	2.8 ± 0.8	2.8 ± 0.9	2.9 ± 0.8	2.7 ± 0.8	3.0 ± 0.9	2.6 ± 0.9	
median (Iqr)	3 (2–3)	3 (2–3) ^5^	3 (2–3) ^3,5^	3(2–3) ^4^	3 (2–4) ^5^	3 (2–3)	
(A.4) How would you score your degree of job satisfaction? Score 0–4							<0.001 §
I’m not satisfied-I’m very satisfied, mean ± sd	2.8 ± 1.0	2.6 ± 1.1	3.0 ± 1.0	2.8 ± 1.1	3.4 ± 0.8	3.0 ± 1.1	
median (Iqr)	3 (2–4)	3 (2–3) ^5^	3 (2–4) ^3,5^	3 (2–4) ^4^	4 (3–4) ^5^	3 (2–4)	
(A.5) What worries you most about your ward/department? *n* (%)							
(A.5.1) Insufficient information about infection							<0.001 #
Very worried	421 (20.26)	158 (18.65)	146 (21.86)	80 (21.39)	24 (25.53)	13 (13.68)	
Quite worried	1038 49.95)	405 (47.82)	350 (52.40)	188 (50.27)	55 (58.51)	40 (42.11)	
Not worried	619 (29.79)	284 (33.53)	172 (25.75)	106 (28.34)	15 (15.96)	42 (44.21)	
(A.5.2) Insufficient personal protective equipment							0.274 #
Very worried	688 (33.11)	285 (33.65)	223 (33.38)	120 (32.09)	38 (40.43)	22 (23.16)	
Quite worried	872 (41.96)	359 (42.38)	278 (41.62)	149 (39.84)	38 (40.43)	48 (50.53)	
Not worried	518 (24.93)	203 (23.97)	167 (25.00)	105 (28.07)	18 (19.15)	25 (26.32)	
(A.5.3) Insufficient staff							<0.001 #
Very worried	558 (26.85)	207 (24.44)	216 (32.34)	78 (20.86)	37 (39.36)	20 (21.05)	
Quite worried	841 (40.47)	331 (39.08)	280 (41.92)	160 (42.78)	34 (36.17)	36 (37.89)	
Not worried	679 (32.68)	309 (36.48)	172 (25.75)	136 (36.36)	23 (24.47)	39 (41.05)	
(A.5.4) Intentional absenteeism in the workplace							<0.001 #
Very worried	212 (10.20)	54 (6.38)	81 (12.13)	48 (12.83)	16 (17.02)	13 (13.68)	
Quite worried	476 (22.91)	138 (16.29)	183 (27.40)	99 (26.47)	32 (34.04)	24 (25.26)	
Not worried	1390 (66.89)	655 (77.33)	404 (60.48)	227 (60.70)	46 (48.94)	58 (61.05)	
(A.5.5) Lack of recognition of my work							<0.001 #
Very worried	118 (5.68)	33 (3.90)	42 (6.29)	32 (8.56)	4 (4.26)	7 (7.37)	
Quite worried	1475 (70.98)	687 (81.11)	442 (66.17)	224 (59.89)	62 (65.96)	60 (63.16)	
Not worried	485 (23.34)	127 (14.99)	184 (27.54)	118 (31.55)	28 (29.79)	28 (29.47)	

# Chi-square of Fisher Exact-Test; § Kruskal-Wallis Test; ^3–5^ Significant differences between this group and the one mentioned by numbers (correction Bonferroni post hoc tests)

**Table 3:** Health care workers’ perceived risk of infection and perceived information about COVID-19 pandemic.

(B) Perceived Risk of Infection and Perceived Information	All *N* = 2078	1. Physicians *N* = 847	2. Nurses *N* = 668	3. Medical staff *N* = 374	4. Health Care Support Staff *N* = 94	5. Administrative Staff *N* = 95	*p*-Value
(B.1) At the beginning of the pandemic, you thought that your risk of being infected was: *n* (%)							0.001 #
low	367 (17.66)	153 (18.06)	114 (17.07)	69 (18.45)	13 (13.83)	18 (18.95)	
medium	481 (23.15)	178 (21.02)	134 (20.06)	110 (29.41)	25 (26.60)	34 (35.79)	
high	1230 (59.19)	516 (60.92)	420 (62.87)	195 (52.14)	56 (59.57)	43 (45.26)	
(B.2) At the beginning of the pandemic, you thought that your risk of being infected was higher: *n* (%)							<0.001 #
in the workplace	1475 (70.98)	687 (81.11)	442 (66.17)	224 (59.89)	62 (65.96)	60 (63.16)	
outside the workplace	118 (5.68)	33 (3.90)	42 (6.29)	32 (8.56)	4 (4.26)	7 (7.37)	
the same in both environments	485 (23.34)	127 (14.99)	184 (27.54)	118 (31.55)	28 (29.79)	28 (29.47)	
(B.3) Currently you think that your risk of being infected is: *n* (%)							<0.001 #
low	527 (25.36)	224 (26.45)	189 (28.29)	67 (17.91)	31 (32.98)	16 (16.84)	
medium	328 (15.78)	112 (13.22)	104 (15.57)	75 (20.05)	13 (13.83)	24 (25.26)	
high	1223 (58.85)	511 (60.33)	375 (56.14)	232 (62.03)	50 (53.19)	55 (57.89)	
(B.4) Currently, you think that your risk of being infected is higher: *n* (%)							<0.001 #
in the workplace	1000 (48.12)	495 (58.44)	275 (41.17)	155 (41.44)	39 (41.49)	36 (37.89)	
outside the workplace	373 (17.95)	125 (14.76)	133 (19.91)	82 (21.93)	18 (19.15)	15 (15.79)	
the same in both environments	705 (33.93)	227 (26.80)	260 (38.92)	137 (36.63)	37 (39.36)	44 (46.32)	
(B.5) Have people close to you contracted the infection? *n* (%)							<0.001
friends	137 (6.58)	43 (5.06)	32 (4.79)	48 (12.80)	5 (5.32)	9 (9.47)	
colleagues	749 (35.98)	309 (36.35)	252 (37.72)	113 (30.13)	36 (38.30)	39 (41.05)	
family	134 (6.44)	44 (5.18)	41 (6.14)	31 (8.27)	11 (11.70)	7 (7.37)	
at least two of the above	409 (19.64)	169 (19.88)	160 (23.95)	55 (14.67)	11 (11.70)	14 (14.74)	
none of the above	653 (31.36)	285 (33.53)	183 (27.40)	128 (34.13)	31 (32.98)	26 (27.37)	
(B.6) I believe I have sufficient information about: score 1–5							
I strongly disagree—I strongly agree							
contamination routes, mean ± nsd	4.12 ± 1.14	4.24 ± 1.09	4.03 ± 1.17	4.16 ± 1.11	3.73 ± 1.38	4.02 ± 1.16	<0.001
median (Iqr)	5 (4–5)	5(4–5) ^2,4^	4 (4–5)	5 (4–5)^4^	4 (2–5)	4 (4–5)	
preventive measures, mean ± nsd	3.95 ± 1.22	4.14 ± 1.18	3.86 ± 1.26	4.02 ± 1.14	3.73 ± 1.42	3.81 ± 1.28	0.095 §
median (Iqr)	4 (4–5)	4 (4–5)	4 (3–5)	4(4–5)	4 (2–5)	4 (3–5)	
symptoms, mean ± nsd	3.97 ± 1.15	4.14 ± 1.09	3.84 ± 1.19	3.95 ± 1.13	3.61 ± 1.31	3.78 ± 1.19	<0.001 §
median (Iqr)	4 (4–5)	4 (4–5) ^2,3,4,5^	4 (3–5)	4 (4–5)	4 (3–5)	4 (3–5)	
treatment, mean ± nsd	3.31 ± 1.20	3.38 ± 1.18	3.29 ± 1.22	3.25 ± 1.17	3.37 ± 1.28	2.98 ± 1.30	0.029 §
median (Iqr)	4 (2–4)	4 (2–4)^5^	4 (2–4)	3 (2–4)	4 (2–4)	3 (2–4)	
prognosis, mean ± nsd	3.35 ± 1.19	3.39 ± 1.17	3.32 ± 1.20	3.31 ± 1.17	3.53 ± 1.26	3.15 ± 1.32	0.150 §
median (Iqr)	4 (2–4)	4 (2–4)	4 (2–4)	4 (2–4)	4 (3–5)	3 (2–4)	
risk factors, mean ± nsd	3.62 ± 1.21	3.64 ± 1.19	3.58 ± 1.23	3.67 ± 1.17	3.68 ± 1.35	3.46 ± 1.30	0.532 §
median (Iqr)	4 (3–5)	4 (3–5)	4 (3–5)	4 (3–5)	4 (3–5)	4 (2–5)	
(B.7) Do you believe that your department is adequately prepared to cope with the emergency due to the infection? Score 0–4							<0.001 §
I strongly disagree—I strongly agree							
mean ± nsd	3.14 ± 1.12	2.98 ± 1.13	3.23 ± 1.09	3.28 ± 1.09	3.27 ± 1.16	3.36 ± 1.09	
*median (Iqr)*	3 (2–4)	3 (2–4) ^2,3,5^	3 (3–4)	3 (3–4)	3 (3–4)	4 (3–4)	
(B.8) Do you believe that your department provides complete and clear information about the management of the infection? Score 0–4							<0.001 §
I strongly disagree—I strongly agree							
mean ± nsd	3.10 ± 1.15	2.93 ± 1.13	3.14 ± 1.15	3.29 ± 1.14	3.23 ± 1.14	3.55 ± 1.18	
median (Iqr)	3 (2–4)	3 (2–4) ^2,3,5^	3 (2–4) ^5^	3 (3–4)	3 (3–4)	4 (3–4)	

# Chi-square of Fisher Exact-Test; § Kruskal-Wallis Test; ^2−5^ Significant differences between this group and the one mentioned by numbers (correction Bonferroni post hoc tests).

**Table 4:** Health care workers’ psychological aspects.

(C) Psychological Profile	All *N* = 2078	Physicians *N* = 847	Nurses *N* = 668	Medical Staff *N* = 374	Health Care Support Staff *N* = 94	Administrative Staff *N* = 95	*p*-Value
(C.1) Do you believe that your department/ward is paying adequate attention to employees’ mental health in this emergency period? Score 0–4							<0.001 §
I strongly disagree—I strongly agree							
mean ± sd	2.57 ± 1.25	2.38 ± 1.21	2.69 ± 1.26	2.68 ± 1.20	2.59 ± 1.35	2.95 ± 1.33	
median (Iqr)	3 (1–3)	2 (1–3) ^2,3,5^	3 (2–4)	3 (2–4)	3 (1–4)	3 (2–4)	
(C.2) Have you suffered from sleep disturbances in the last two months? *n* (%)							<0.001 #
no	760 (36.57)	320 (37.78)	198 (29.64)	163 (43.58)	33 (35.11)	46 (48.42)	
yes	1318 (63.43)	527 (62.22)	470 (70.36)	211 (56.42)	61 (64.89)	49 (51.58)	
(C.3) Do you believe psychological support is useful in the current situation? *n* (%)							0.016 #
no	346 (16.65)	137 (16.17)	99 (14.82)	67 (17.91)	15 (15.96)	28 (29.47)	
yes	1732 (83.35)	710 (83.83)	569 (85.18)	307 (82.09)	79 (84.04)	67 (70.53)	
(C.4) Have you received psychological support in the last two months? *n* (%)							0.092 #
no	1883 (90.62)	757 (89.37)	600 (89.82)	347 (92.78)	88 (93.62)	91 (95.79)	
yes	195 (9.38)	90 (10.63)	68 (10.18)	27 (7.22)	6 (6.38)	4 (4.21)	
(C.5) Does your department/ward provide a psychological support service? *n* (%)							<0.001 #
no	793 (38.16)	358(42.27)	193(28.89)	358 (42.27)	193 (28.89)	22 (23.16)	
yes	1285 (61.84)	489 (57.73)	475 (71.11)	203 (54.28)	45 (47.87)	73 (76.84)	
(C.6) Have you taken psychopharmacological drugs?							0.003 #
no	1848 (88.93)	731 (86.30)	593 (88.77)	350 (93.58)	87 (92.55)	87 (91.58)	
yes	230 (11.07)	116 (13.70)	75 (11.23)	24 (6.42)	7 (7.45)	8 (8.42)	

# Chi-square of Fisher Exact-Test; § Kruskal-Wallis Test; ^2,3,5^ Significant differences between this group and the one mentioned by numbers (correction Bonferroni post hoc tests).

## References

[1] Mukherjee S. (2017). Emerging Infectious Diseases: Epidemiological Perspective. Indian J. Dermatol..

[2] World Health Organization Regional Office for South-East Asia A Brief Guide to Emerging Infectious Diseases and Zoonoses. https://apps.who.int/iris/handle/10665/204722%0A.

[3] Belay E.D., Kile J.C., Hall A.J., Barton-Behravesh C., Parsons M.B., Salyer S., Walke H. (2017). Zoonotic disease programs for enhancing global health security. Emerg. Infect. Dis..

[4] Rabozzi G., Bonizzi L., Crespi E., Somaruga C., Sokooti M., Tabibi R., Vellere F., Brambilla G., Colosio C. (2012). Emerging Zoonoses: The “One Health Approach”. Saf. Health Work.

[5] Nii-Trebi N.I. (2017). Emerging and Neglected Infectious Diseases: Insights, Advances, and Challenges. Biomed. Res. Int..

[6] Jones K.E., Patel N.G., Levy M.A., Storeygard A., Balk D., Gittleman J.L., Daszak P. (2008). Global trends in emerging infectious diseases. Nature.

[7] Yeh H.-Y., Chen K.-H., Chen K.-T. (2018). Environmental Determinants of Infectious Disease Transmission: A Focus on One Health Concept. Int. J. Environ. Res. Public Health.

[8] Institute of Medicine (US) Forum on Microbial Threats (2009). Infectious Disease Emergence: Past, Present, and Future. Microbial Evolution and Co-Adaptation.

[9] Jiang L., Ng I.H.L., Hou Y., Li D., Tan L.W.L., Ho H.J.A., Chen M.I.C. (2018). Infectious disease transmission: Survey of contacts between hospital-based healthcare workers and working adults from the general population. J. Hosp. Infect..

[10] Suwantarat N., Apisarnthanarak A. (2015). Risks to healthcare workers with emerging diseases. Curr. Opin. Infect. Dis..

[11] Ruotsalainen J.H., Verbeek J.H., Mariné A., Serra C. (2015). Preventing occupational stress in healthcare workers. Cochrane Database Syst. Rev..

[12] Koinis A., Giannou V., Drantaki V., Angelaina S., Stratou E., Saridi M. (2015). The impact of healthcare workers job environment on their mental-emotional health—Coping strategies: The case of a local general hospital. Heal. Psychol. Res..

[13] Tan B.Y.Q., Chew N.W.S., Lee G.K.H., Jing M., Goh Y., Yeo L.L.L., Zhang K., Chin H.-K., Ahmad A., Khan F.A. (2020). Psychological Impact of the COVID-19 Pandemic on Health Care Workers in Singapore. Ann. Intern. Med..

[14] Goulia P., Mantas C., Dimitroula D., Mantis D., Hyphantis T. (2010). General hospital staff worries, perceived sufficiency of information and associated psychological distress during the A/H1N1 influenza pandemic. BMC Infect. Dis..

[15] Kabene S.M., Orchard C., Howard J.M., Soriano M.A., Leduc R. (2006). The importance of human resources management in health care: A global context. Hum. Resour. Health.

[16] WHO COVID-19 Disrupting Mental Health Services in Most Countries: WHO Survey. https://www.who.int/news/item/05-10-2020-covid-19-disrupting-mental-health-services-in-most-countries-who-survey.

[17] Coronavirus Disease (COVID-19). https://www.who.int/emergencies/diseases/novel-coronavirus-2019.

[18] Puci M.V., Loi F., Ferraro O.E., Cappai S., Rolesu S., Montomoli C. (2020). COVID-19 trend estimation in the elderly Italian region of Sardinia. Front. Public Health.

[19] Marazziti D., Stahl S.M. (2020). The relevance of COVID-19 pandemic to psychiatry. World Psychiatry.

[20] Remuzzi A., Remuzzi G. (2020). COVID-19 and Italy: What next?. Lancet.

[21] Italian President of the Council of Ministers (2020). Decree of 17 March 2020.

[22] Nguyen L.H., Drew D.A., Graham M.S., Joshi A.D., Guo C.-G., Ma W., Mehta R.S., Warner E.T., Sikavi D.R., Lo C.-H. (2020). Risk of COVID-19 among front-line health-care workers and the general community: A prospective cohort study. Lancet Public Health.

[23] Lai J., Ma S., Wang Y., Cai Z., Hu J., Wei N., Wu J., Du H., Chen T., Li R. (2020). Factors Associated With Mental Health Outcomes Among Health Care Workers Exposed to Coronavirus Disease 2019. JAMA Netw. Open.

[24] Lai X., Wang M., Qin C., Tan L., Ran L., Chen D., Zhang H., Shang K., Xia C., Wang S. (2020). Coronavirus Disease 2019 (COVID-2019) Infection Among Health Care Workers and Implications for Prevention Measures in a Tertiary Hospital in Wuhan, China. JAMA Netw. Open.

[25] Brug J., Aro A.R., Richardus J.H. (2009). Risk perceptions and behaviour: Towards pandemic control of emerging infectious diseases: International research on risk perception in the control of emerging infectious diseases. Int. J. Behav. Med..

[26] Garzaro G., Clari M., Ciocan C., Grillo E., Mansour I., Godono A., Borgna L.G., Sciannameo V., Costa G., Raciti I.M. (2020). COVID-19 infection and diffusion among the healthcare workforce in a large university-hospital in Northwest Italy. Med. Lav..

[27] Nyblade L., Stockton M.A., Giger K., Bond V., Ekstrand M.L., Lean R.M., Mitchell E.M.H., Nelson L.R.E., Sapag J.C., Siraprapasiri T. (2019). Stigma in health facilities: Why it matters and how we can change it. BMC Med..

[28] Knaak S., Mantler E., Szeto A. (2017). Mental illness-related stigma in healthcare. Healthc. Manag. Forum.

[29] Fowler F. (2009). Survey Research Methods.

